# Hexadecylamine Addition Promotes Crystallization-Driven Functionalities in Freezing–Thaw PVA Hydrogels

**DOI:** 10.1021/acs.macromol.5c01534

**Published:** 2025-11-28

**Authors:** Alexis Alvear-Jiménez, Mercedes Fernández, Alejandro J. Müller, Rebeca Hernández

**Affiliations:** † Institute of Polymer Science and Technology ICTP-CSIC, Juan de la Cierva 3, 28006 Madrid, Spain; ‡ POLYMAT and Department of Polymers and Advanced Materials: Physics, Chemistry and Technology, Faculty of Chemistry, 160665 University of the Basque Country UPV/EHU, Paseo Manuel de Lardizabal, 3, 20018 Donostia-San Sebastián, Spain; § IKERBASQUE, Basque Foundation for Science, Plaza Euskadi 5, 48009 Bilbao, Spain

## Abstract

Poly­(vinyl alcohol) (PVA) is widely employed for hydrogel fabrication due to its ability to form stable, physically or chemically cross-linked three-dimensional networks. Among production methods, the freezing–thaw (F–T) technique stands out for its simplicity and effectiveness. This study presents a straightforward methodology for preparing functional PVA hydrogels in aqueous media by blending PVA with a crystallizable hexadecyl alkylamine (C16). Incorporating small amounts of hydrophobic, amphiphilic hexadecylamine into PVA hydrogels via freezing–thaw blending significantly alters crystallization and network structure, enabling gel formation in water without surfactants, solvents, or cross-linkers. C16 enhances phase separation and promotes PVA crystalline domains while remaining amorphous. The resulting PVA/C16 hydrogels exhibit enhanced thermomechanical properties, demonstrating additional functionalities such as self-healing and improved adhesion to polar surfaces. This crystallization-driven gelation via immiscible blending offers a scalable strategy with potential in wearable electronics, soft robotics, and biomedical devices.

## Graphical abstract

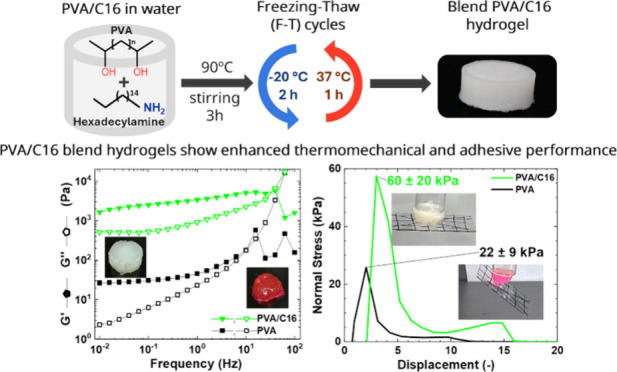

## 1 Introduction

Poly­(vinyl alcohol) (PVA) is widely employed to fabricate hydrogels because it can form chemically or physically cross-linked stable three-dimensional networks. These networks provide a porous structure that can be used in drug release, as a substrate for cell and tissue culture, or water treatment, among other applications. Earlier studies in literature showed that it is possible to obtain PVA hydrogels, also known as PVA cryogels, through freezing-thaw (F-T) cycles of aqueous solutions of PVA. In this method, an aqueous solution of PVA with concentrations ranging between 2.5–15 wt % is frozen to temperatures below −20 °C and subsequently thawed back to room temperature for gel formation.^1^

Over the past few decades, significant attention has been paid to elucidating the structural organization of F-T PVA hydrogels through various experimental techniques. There is general agreement that F-T PVA hydrogels exhibit a porous structure due to water crystallization within the pores (when the samples are subjected to the freezing part of the F-T cycles) occupied by water, along with domains containing crystalline aggregates and amorphous PVA chains swollen by the solvent.^2–6^ The possibility of chemical cross-linking resulting from the formation of PVA radicals due to the ice shearing of PVA chains has also been highlighted as a potential mechanism for gel formation.^7^ Since the pioneering work of Peppas et al., the freezing and thawing method has been extensively investigated for gel formation in various applications, and a recent report outlines fundamental knowledge and recent applications of F-T PVA hydrogels.^8^

Interestingly, F-T PVA hydrogels have been reported to exhibit self-healing properties in the absence of any external healing agent or stimuli due to the formation of hydrogen bonding over time in gel pieces with sufficient hydroxyl groups or high chain mobility; this property is highly dependent on crystallite size. The formation of large crystallites hinders chain diffusion and/or reentanglement formation.^9,10^

Crystallization-induced self-healing in polymer hydrogels involves incorporating crystallizable alkyl chains that enable self-healing when cooling the gel specimen below the crystallization temperature of the side chains. Most previous studies on incorporating long alkyl side groups into hydrophilic polymer backbones for crystallization-induced gel formation have relied on the micellar copolymerization of hydrophilic and hydrophobic monomers in the presence of surfactants like sodium dodecyl sulfate (SDS). To date, most reported hydrogels consist of polyacrylamide or poly­(acrylic acid) chains with high levels of crystallizable octadecyl acrylate segments, typically synthesized with a chemical cross-linker.^11^ As a network cross-linker class with stable swelling behavior, the hydrophobic association of alkyl side chains has been widely used to develop tough hydrogels with shape-memory functions (e.g., semicrystalline hydrogels). These side chains must be sufficiently long to form crystals, typically containing more than 11 carbon atoms.^12^

Other approaches have explored the hydrophobic modification of PVA for gel formation.^13^ However, when PVA is substituted with hexyl-carbamate side chains (DS ∼ 50%), gel formation occurs in organic solvents such as dimethylformamide (DMF) and dimethyl sulfoxide (DMSO), as these highly substituted PVAs are not water-soluble. Consequently, gelation must be achieved via solvent–nonsolvent exchange.^12^ Preparing PVA hydrogels with strong mechanical properties typically involves using organic solvents when PVA is modified with crystallizable side chains or multiple F-T cycles to promote sufficient crystallization among polymer chains.^14,15^

Recent studies have explored adding secondary compounds to modify the crystallinity of PVA hydrogels formed through F-T cycles, leading to new formulations. For example, incorporating phytic acid as a gelator enhances hydrogen bonding between PVA chains, weakening PVA crystallization and thereby increasing the hydrogel’s mechanical properties and optical transparency.^16,17^ In contrast, other studies have reported the opposite effect, where increased mechanical properties were achieved by enhancing PVA crystallinity by adding a secondary compound, which promoted higher PVA concentration in the crystalline regions of the gel.^18^

This work presents a straightforward methodology for forming PVA gels in water by blending PVA with a crystallizable hexadecyl alkylamine (C16) and using freezing–thaw (F-T) cycles to facilitate gelation. This simple system, in which gel formation occurs exclusively in water, demonstrates that the incorporation of a second, immiscible crystallizable component within the hydrogel—even in small amounts—can significantly modify the thermomechanical properties of the resulting hydrogels, introducing additional functionalities such as self-healing and adhesive properties.

## 2 Experimental Section

### 2.1 Materials

A commercially available PVA, 99.3–100% hydrolyzed with an average molecular weight in the range of 146–186 kg/mol and 1-Hexadecylamine (with a molar mass of 241.5 g/mol), 90%, were purchased from Sigma-Aldrich. Compounds were used without any prior treatment.

### 2.2 Hydrogels Preparation

Aqueous 10% (w/w) PVA solutions were prepared in Milli-Q water by dissolving PVA overnight at 90 °C. Subsequently, 1-hexadecylamine was incorporated at concentrations ranging from 0% to 4% (w/w). The resulting solutions were stirred for 12 h at 90 °C. Subsequently, a sonicator was used to eliminate bubbles trapped within the solution. The final solution was poured into molds to obtain specimens measuring 1 cm in height and 2 cm in diameter and subjected to 4 F-T cycles to produce the hydrogels, consisting of a 2-h freezing step performed at −20 °C in a freezer, followed by a 1-h thawing step performed at 37 °C in an oven (Figure 1). The resulting PVA/1-hexadecylamine blend hydrogels were designated as PVA/C16_X “as prepared” hydrogels, where X is the solid content of C16 in the hydrogel. A second series of samples was prepared, in which “partially dried” hydrogels were obtained by maintaining the “as prepared” hydrogels at *T* = 37 °C for 2 h. Finally, fully dried samples were produced through freeze-drying. For comparison, neat PVA hydrogels with no added C16 were also prepared following the same experimental protocol.

**Figure 1:**
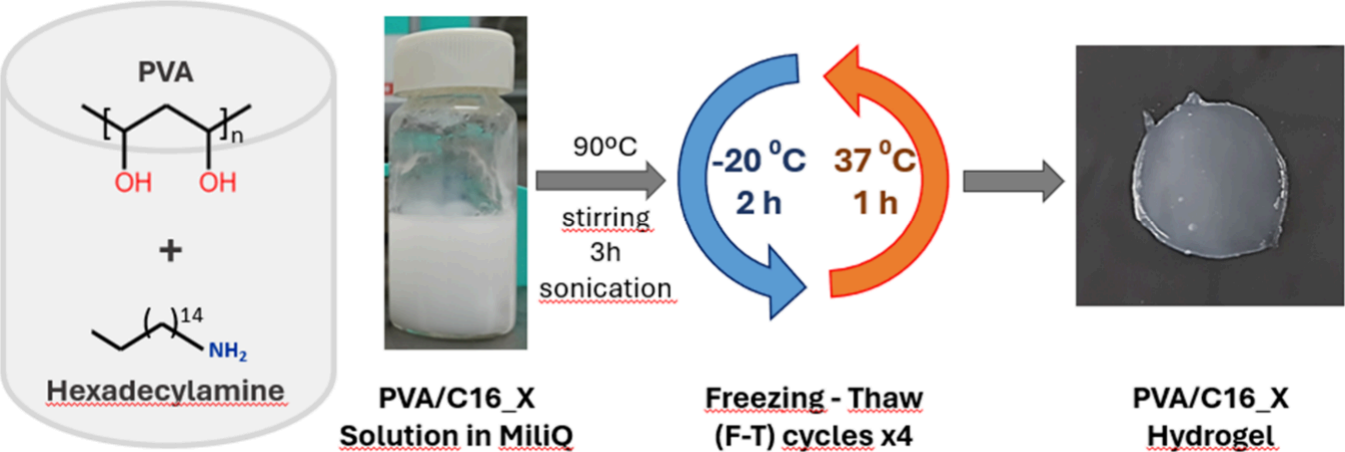
Experimental procedure for the preparation of PVA/C16_X blend hydrogels.

The solid content (SC) of “as prepared” and “partially dried” hydrogels was calculated as the weight of the lyophilized hydrogel divided by the total weight of the hydrogel.

### 2.3 Raman Spectroscopy

Raman measurements were performed at the Raman Microspectroscopy Laboratory of the Characterization Service in the Institute of Polymer Science & Technology (CSIC) using a Renishaw InVia-Reflex system (Renishaw plc, UK). The instrument features a grating spectrometer with a Peltier-cooled CCD detector coupled to a confocal microscope. An argon ion laser (λ = 514.5 nm) provided excitation, and a 100× objective (NA = 0.85) delivered approximately 2 mW of laser power to the sample. Spectra were collected at several locations from freeze-dried samples.

### 2.4 Differential Scanning Calorimetry (DSC)

Calorimetric analyses were conducted using a TA Instruments Q100 differential scanning calorimeter linked to a cooling system under dry nitrogen (50 cm^3^/min). All measurements were done at a constant rate of 5 °C/min. Both the hydrogels and the freeze-dried samples were sealed in aluminum pans. The first DSC heating scans for these samples were carried out from 20 to 90 °C. The degree of crystallinity, *X*_c_ (DSC), of the PVA hydrogels and their blend hydrogels with C16 was determined as the ratio between the experimentally determined heat of fusion, Δ*H*_m_, of the PVA hydrogel sample and the enthalpy of melting of a 100% crystalline PVA, Δ*H*_m_° = 150 J/g:^19^

$$X_{c}(\mathrm{DSC})=(\Delta H_{\exp}/\Delta H_{m}^{o}\times f)\times 100$$ 1

Where *f* is the fraction of PVA in the sample.

### 2.5 Wide-Angle X-ray Scattering

Wide-angle X-ray scattering (WAXS) measurements were conducted at beamline BL11-NCD of the ALBA Synchrotron in Barcelona, Spain. The samples were contained in DSC aluminum pans, and a Linkam THMS600 hot stage coupled with a liquid nitrogen cooling system was used to heat the samples at a rate of 5 °C/min. A 12.4 keV (λ = 1.03 Å) X-ray energy source was utilized. The distance between the sample and the detector (ADSC Q315r detector, Poway, CA, USA, with a resolution of 3070 × 3070 pixels and a pixel size of 102 μm^2^) was 92.1 mm with a tilt angle of 21.2°. Calibration was conducted using chromium­(III) oxide (Rayonix LX255-HS detector, Evanston, IL, USA, with a resolution of 1920 × 5760 pixels and a pixel size of 44 μm^2^). Plots of scattering intensity as a function of the scattering vector were obtained for WAXS data, where the scattering vector is defined as *q* = 4π (sin θ)/λ, with λ representing the X-ray wavelength and 2θ the scattering angle.

### 2.6 Characterization of the Self-Healing and Mechanical Properties

Optical evidence was gathered to characterize the self-healing performance of the samples under study. The prepared hydrogels, both with and without rhodamine B (added to provide color to the hydrogels), were cut into two halves by the middle. The two separate pieces of hydrogel were then placed in contact for self-healing in a container of water at 37 °C without any external stimulus or the addition of healing agents. Subsequently, the same experiment was carried out in a container of water maintained at *T* = 60 °C for 24 and 72 h. The rheological properties of “as prepared” hydrogels were compared before and after the thermal treatment at 60 °C for 72 h in water. Rheological oscillatory sweeps were performed using an AR-G2 rheometer (TA Instruments, USA) with a 20 mm plate–plate geometry and a solvent trap to prevent water evaporation. Oscillatory temperature sweeps were conducted at a frequency of 1 Hz within the temperature range of 20 to 90 °C, with a heating rate of 5 °C/min. Frequency sweeps were conducted from 0.01 to 100 Hz. All measurements were performed at a constant strain of 1% within the linear viscoelastic range. The results were analyzed using the TRIOS software from TA Instruments.

### 2.7 Determination of Adhesive Properties

The adhesion capacity was tested by visual experiments carried out for “as prepared” hydrogels maintained at *T* = 60 °C for 72 h on five different surfaces**:** skin, cardboard, rubber, glass, and polypropylene. The adhesion force was measured using an ARES strain-controlled rheometer (TA Instruments) equipped with an 8 mm glass tack probe, which matched the sample’s diameter, resulting in a contact area of 50.3 mm^2^ between the upper and lower plates. In each case, a hydrogel sample with a thickness of 500 μm was positioned on the lower plate of the rheometer. The probe-tack test was performed in three stages: (1) compression in which the upper plate descended at 0.1 mm/s until a material layer of *h*_0_ = 50 μm was formed, (2) contact period for 60 s, and (3) debonding at (*V*_deb_) 0.314 mm/s. Force (*F*)–displacement (*h*(*t*)) data were acquired during each experiment and transformed to Normal stress (kPa)–Strain (−) curves.

## 3 Results and Discussion

### 3.1 Gel Formation

Gel formation was confirmed for PVA/C16 blend hydrogels at C16 contents in the range between 1 and 3 wt % after undergoing 4 F-T cycles following the experimental procedure depicted in Figure 1. C16 is insoluble in water and forms white opaque solutions when added to 10% (w/w) PVA aqueous solutions, as shown in Figure S1 of the Supporting Information, Supporting Information. At C16 concentrations above 4 wt %, precipitates were observed, indicating a nonhomogeneous dispersion that leads to phase separation and precipitation in solution hence preventing gel formation. The solid content of the “as prepared” hydrogels increased from 10% w/w for the neat PVA hydrogel (without the addition of C16) to 13% w/w for the PVA/C16_3 sample. The “partially dried” hydrogels elevated the solid content to approximately 20% w/w for all samples Table 1 thus indicating that the water content in the samples remains unaffected by the C16 concentration.

**Table 1:** Composition and solid content of the samples under study

	“as prepared” hydrogels			“partially dried” hydrogels	lyophilized	
	PVA	C16	PVA+C16	PVA+C16	PVA	C16
sample	%	%	%	%	%	%
**PVA**	10	-	10	19.9 ± 1.5	100	-
**PVA/C16_1**	10	1	11	18.1 ± 0.8	90.9	9.1
**PVA/C16_2**	10	2	12	21.5 ± 4.4	83.3	16.7
**PVA/C16_3**	10	3	13	20.7 ± 1.2	76.9	23.1

As seen in figure (Figure 2a), both the neat PVA hydrogels and the PVA/C16 blend hydrogels have an opaque appearance, which may be due to the formation of phase-separated domain sizes resulting from F-T cycles. As previously reported, F-T PVA hydrogels exhibit a phase-separated porous structure, with pores filled by a polymer-poor phase. The network scaffolding is maintained by highly interconnected regions of a polymer-rich phase. This phase is itself organized and consists of small crystalline aggregates of PVA chains and amorphous domains.^8,20–22^

**Figure 2:**
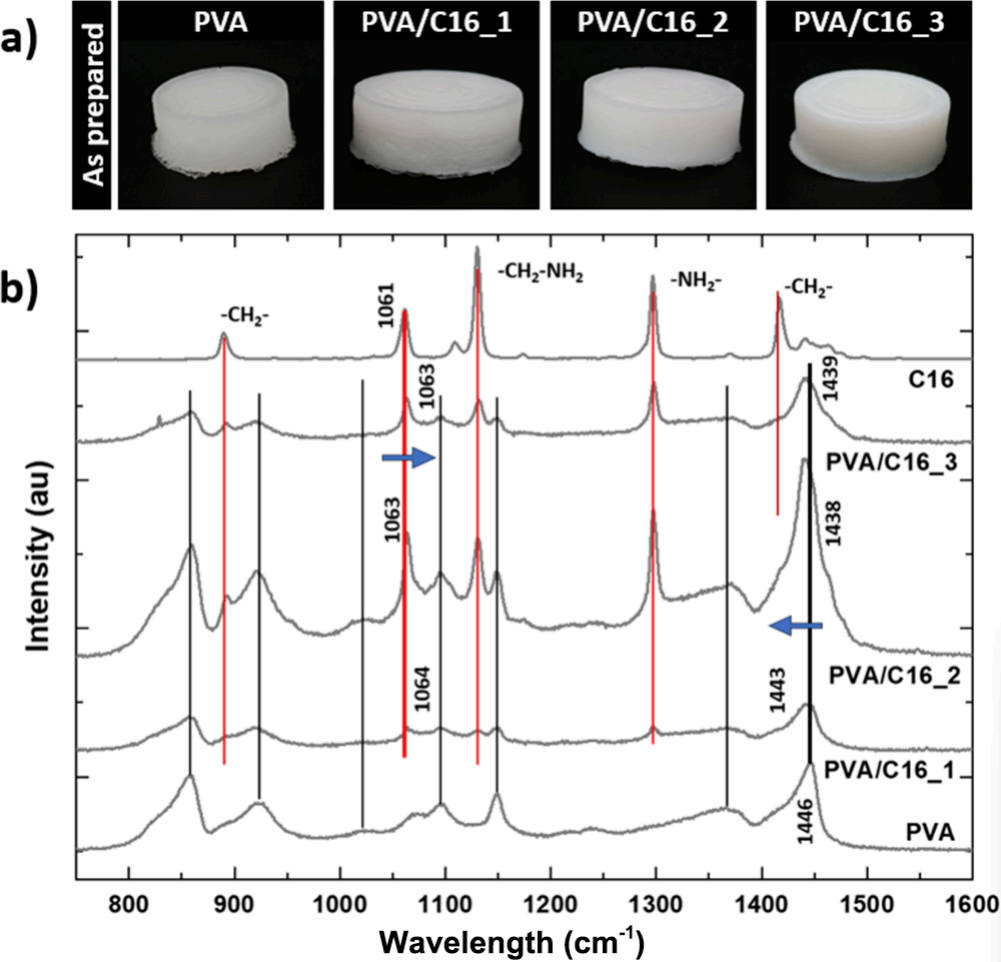
(a) Macroscopic appearance of “as prepared” neat PVA hydrogels and PVA/C16 blend hydrogels. (b) Raman spectra corresponding to freeze-dried samples. For comparison, the Raman spectrum of C16 is also shown. Black lines mark the position of the characteristic bands of PVA, and red lines mark the position of the characteristic bands of C16. For easier visualization, blue arrows mark the shifting of characteristic bands of PVA and C16 in blend hydrogels.

To determine the presence of interactions between PVA and C16, Raman spectroscopy was carried out on lyophilized hydrogels, and the results are shown in (Figure 2b).

The PVA spectrum features two bands associated with −OH bending at 1096 and 1446 cm^–1^. The C–C stretching is observed at 857, 923, and 1149 cm^–1^, while the C–O stretching appears at 1024 and 1149 cm^–1^. The C16 spectrum exhibits characteristic bands at 890 cm^–1^, which can be attributed to −CH_2_ rocking or NH_2_ wagging, and at 1061 and 1130 cm^–1^, corresponding to C–N stretching. The band at 1296 cm^–1^ is associated with the NH_2_ twisting mode, whereas the CH_3_ deformation vibration associated with amine group interaction is shown at 1416 cm^–1^.^22^ PVA/C16 blend hydrogels present the characteristic bands for both C16 and PVA. Compared to the neat PVA hydrogel, for blend hydrogels, the band located at 1446 cm^–1^ in PVA hydrogels gradually shifted to lower wavenumbers with the C16 content until 1439 cm^–1^ for the PVA/C16_3 sample. In addition, the band located at 1061 cm^–1^ in C16 slightly shifted toward higher wavenumbers. This red shift of the OH groups suggested the enhancement of the hydrogen bonding, which led to the formation of hydrogen-bonded abundant regions that had previously been related to increased crystallinity and might also indicate the presence of interactions with the amine groups of C16.^23^ In fact, ATR-FTIR spectra reported in (Figure S2, Supporting Information), showed a shift of the band located at 1540 cm^–1^ assigned to N–H bending from 1542 cm^–1^ in C16 to 1570 cm^–1^ for PVA blend hydrogels, which further support the existence of interactions involving C16 amine groups.^24^

### 3.2 Crystallinity in PVA/C16 Blend Hydrogels

The thermal behavior of both neat PVA hydrogels and PVA/C16 blend hydrogels was investigated using DSC, with experiments performed on both “as prepared” and “partially dried” samples. The DSC experiments performed on neat PVA hydrogels, both in the “as prepared” state (Figure 3a) and after partial drying (Figure 3b), revealed no detectable thermal transitions in the explored temperature range, neither crystallization nor melting. This suggests that the samples are amorphous or that the crystallinity is so low that DSC cannot detect it in the hydrogel samples. Indeed, WAXS measurements (Figure 4 below) on similar neat PVA hydrogel samples detected a very weak reflection (at approximately *q* = 13.8 nm^–1^) that corresponds to diffraction from the (101)/(101̅) crystallographic planes of PVA.^25,26^ Such low crystallinity levels are consistent with previous reports in the literature, where PVA hydrogels thermally treated by F-T cycles exhibited only a small degree of crystallinity. As an example, Ricciardi et al.^19^ reported that the crystallinity of PVA hydrogels increased from approximately 1.7 to 4.7% as the number of F-T cycles increased, reaching a plateau after 3–5 cycles. This low crystallinity is likely a consequence of the F-T process, which generates a physically cross-linked hydrogel structure but does not promote significant crystallization on neat PVA.

**Figure 3:**
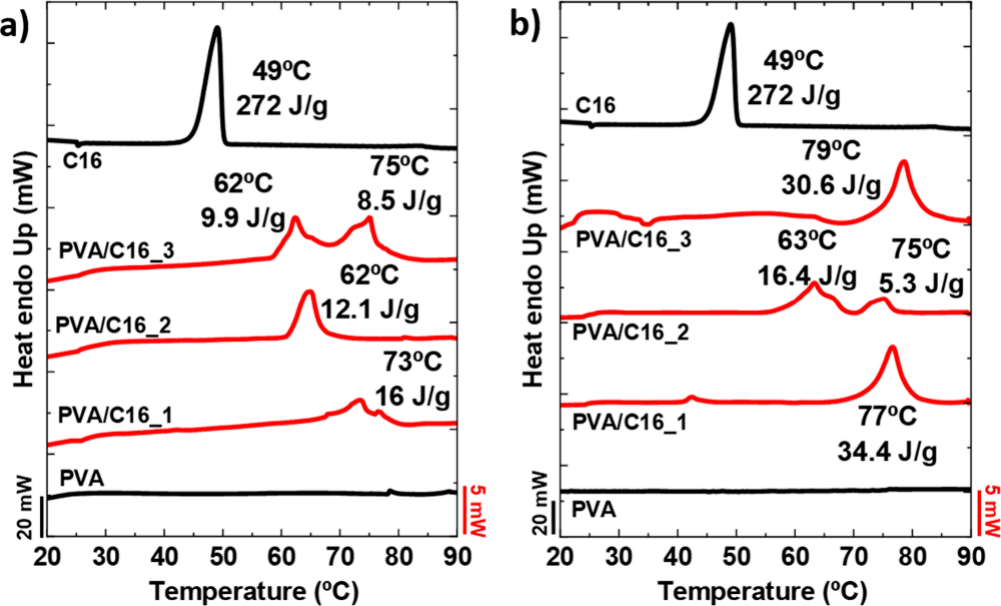
First DSC heating scan corresponding to (a) “as prepared” hydrogels and (b) “partially dried” hydrogels obtained after 2 h at *T* = 37 °C. The Δ*H*_m_ values of the transition peaks for “as prepared” and “partially dried” are normalized by the weight of PVA in the gel. The results corresponding to neat bulk C16 are also depicted for each curve set as a reference.

**Figure 4:**
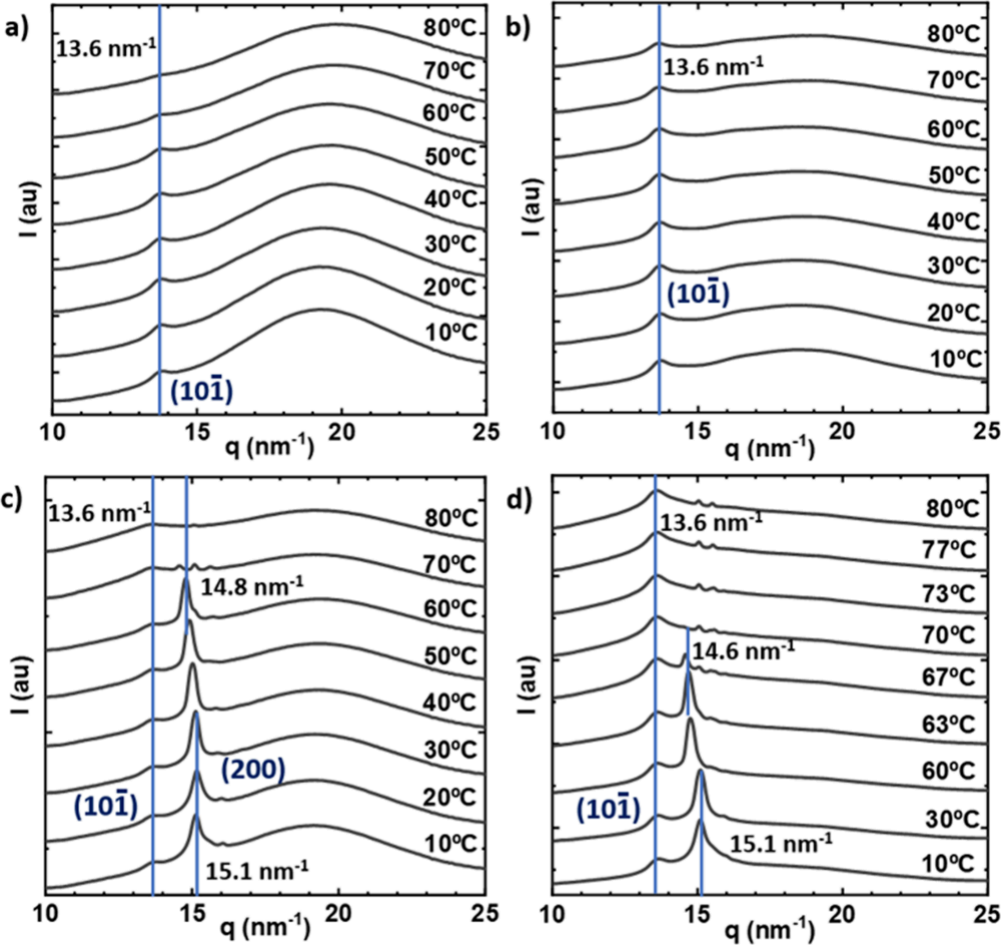
WAXS patterns obtained during the heating at 1 °C/min with different water content. Neat PVA hydrogels (a) “as prepared” and (b) “partially dried”. Sample PVA/C16_3 blend hydrogels (c) “as prepared” and (d) “partially dried”.

In contrast to the neat PVA hydrogels, the first DSC heating scan of the “as prepared” PVA/C16 blend hydrogels revealed relatively broad (and sometimes bimodal) endothermic peaks in the temperature range of 55–80 °C for all C16 concentrations (Figure 3a). These peaks correspond to the melting of PVA crystals formed within the hydrogel network, as confirmed by WAXS experiments to be discussed below (Figure 4c,d). WAXS did not detect any signals that correspond to C16 crystals. The low-temperature PVA melting range in the blend gels (55–80 °C) is caused by the melting point depression induced by the water content in the gel. As a bulk semicrystalline polymer, the melting temperature, *T*_m_, of PVA typically ranges from 180 to 240 °C, depending on its degree of hydrolysis and tacticity.^27^ Despite the lower melting point of the PVA crystals within the blend hydrogels, their melting temperature occurs at higher temperatures than the melting of bulk C16 crystals (i.e., 49 °C), shown in Figure 3 for comparison purposes. This is also consistent with the endothermic transitions in Figure 3 being due exclusively to the fusion of PVA crystals (as supported by WAXS evidence). The presence of PVA crystal melting in these blend samples suggests that adding C16 promotes the formation of PVA crystalline regions in the hydrogel. This novel and interesting result is likely due to the amphiphilic nature of hexadecylamine, which enhances phase separation from PVA chains, thus promoting the formation of PVA crystals. The lack of crystallization and melting of the C16 chains is likely due to their small amount in the blends and their interactions with PVA chains, as demonstrated by Raman spectroscopy (Figure 2b).

The broad and complex endothermic melting processes (sometimes displaying two distinct peaks and shoulders within the main melting peaks) observed in the blend hydrogels of Figure 3a may result from the intricate crystal morphology formed during the F-T cycles. It is possible that crystal aggregates with different lamellar thicknesses develop depending on their specific location within the hydrogel. As is well-known, the melting point depends on lamellar thickness, as predicted by the Gibbs–Thomson equation. Furthermore, part of the observed endothermic processes may also be due to the gel–sol phase transition, where the melting of the crystals creates an endothermic peak, followed by the solubilization and solvation of the polymer chains, which produce an exothermic peak, even when the melting process is not complete. Therefore, there is an overlap between the melting and the solubilization of the PVA chains. This may also cause uncertainties in the crystallinity degrees determined by DSC.

The first DSC heating scan corresponding to “partially dried” PVA/C16 blend hydrogels (Figure 3b) showed higher enthalpy values when compared to “as prepared” PVA/C16 blend hydrogels. This result suggests that drying increases the crystallinity of the hydrogel, as water removal increases PVA concentration and phase separation, allowing more PVA chains to crystallize. Interestingly, although the degree of crystallinity initially increased during drying, it showed a slight decreasing trend as the C16 content increased, dropping from 15.1% to 12.5%. It is important to note that part of the endothermic transitions observed in Figure 3 result from the overlap between polymer solubilization and PVA crystal melting, which may lead to an underestimation of crystallinity. This explains why an apparent decrease in PVA crystallinity was observed in these partially dried blend gels. This phenomenon has been previously reported in *aged* F-T PVA hydrogels, and it has been attributed to the gradual restructuring of the hydrogel network upon heating, where polymer chains undergo solvation before complete melting.^19^

The results obtained from DSC provide evidence that PVA/C16 blend hydrogels exhibit enhanced phase separation and increased PVA crystallinity compared to neat PVA hydrogels. To gain a deeper understanding of these structural changes, WAXS measurements were conducted as a function of temperature.

The WAXS patterns for the “as prepared” PVA/C16_3 blend hydrogel (Figure 4c), displayed a diffraction peak at *q* = 13.6 nm^–1^, similar to that observed in neat PVA hydrogels, which could be assigned to the (101̅) reflection of PVA crystals that could also be overlapped with the (101) reflection as previously reported in literature.^25,26^ However, in contrast to the neat PVA hydrogels, an additional strong scattering peak appeared at *q* = 15.1 nm^–1^ (at 10 °C), which could be tentatively attributed to the (200) reflection of PVA crystals. For semicrystalline PVA, the (200) reflection typically appears at around *q* ≈ 16 nm^–1^, as reported in the literature.^28,29^ In this work, reflections attributed to the (200) planes have been observed for the sample PVA/C16_3 blend hydrogel in the range of 14.6 to 15.1 nm^–1^, depending on the temperature (Figure 4c,d). If PVA WAXS spectra from the literature are examined, there are no other reflections in this *q* range. Since the shift in *q* is rather small, we speculate that the reflection could be due to the X-ray scattering from the (200) planes. This is the reason why we have indexed this reflection as due to the (200) planes in Figure 4c,d. A shift to lower *q* values could indicate an expansion of the PVA unit cell along the *a*-axis. Increases in unit cell dimensions and the *a*-axis have been reported in the literature as the temperature of bulk neat PVA increased.^28^

The change in *q* values from 10 °C (15.1 nm^–1^) to 68 °C (14.6 nm^–1^) can be attributed to the increase in temperature during the measurements (see Figure 4d). It should be noted that the observed reflections in the range 14.6 to 15.1 nm^–1^ must be due to PVA crystals, as they do not disappear at temperatures beyond 50 °C (temperatures that are higher than the melting point of neat bulk C16 crystals) and instead vanish at temperatures above 80 °C, coinciding with the measurements that we report by DSC in Figure 3b.

The reason why the strong reflection attributed to the (200) plane appears slightly shifted to lower *q* values compared to the literature is unknown. One could speculate that the shift is caused by the presence of C16 chains in the blend hydrogel, which interact with the PVA chains, as indicated by our Raman results (Figure 2b). Another possibility is the inclusion of water in the PVA unit cells, since this phenomenon has also been observed when neat PVA is exposed to humidity.^28^ However, a more detailed study, outside the scope of the present paper, would be necessary to determine the exact reasons for this change.

The *q* = 13.6 nm^–1^ peak (corresponding to the (101̅) reflection does not change much in relative intensity when C16 is added to PVA. It must be considered that this peak is a minor reflection, and changes in its intensity could be beyond the resolution of the experiments. Similar results with insignificant changes in this (101̅) reflection have been reported in the literature, along with the appearance of the (200) additional reflection leading to an increase in the degree of crystallinity in PVA hydrogels.^30^ The presence of the strong (200) additional reflection in Figure 4c suggests that the incorporation of C16 induces the crystallization of PVA in PVA/C16 blend hydrogels, promoting phase separation and increasing the amount of PVA crystals that can be formed. Both diffraction peaks disappeared above 80 °C, in consonance with the DSC results (see Figure 3b).

For “partially dried” PVA/C16_3 blend hydrogels (Figure 4d), the same diffraction peaks at *q* = 13.6 nm^–1^ and *q* = 14.7 nm^–1^ were observed, but with increased intensity. This enhancement in diffraction peak intensity is attributed to the lower water content in these samples, which allows for a higher degree of crystallinity. A direct comparison between the WAXS diffractograms of neat PVA hydrogels and PVA/C16 blend hydrogels confirms that the presence of C16 leads to a significant increase in crystallinity. This finding is in full agreement with the DSC results shown in Figure 3, where the enthalpy of fusion (Δ*H*_m_) and the degree of crystallinity (*X*_c_) of the PVA component were shown to greatly increase in PVA/C16 blend hydrogels compared to neat PVA hydrogels (which by DSC appeared amorphous).

### 3.3 Crystallinity-Driven Functionality: Self-Healing and Adhesive Behavior

Hydrogels can obtain thermal energy and regulate the polymer network (hydrogen bonding or water content) through heating, contributing to thermal-responsive self-healing. In particular, such characteristics have been reported for PVA hydrogels fabricated by the F-T process for PVA concentrations above 25 wt %.^8^ For the samples under study herein, the PVA concentration was maintained at 10 wt %, and none of the samples showed self-healing when kept at room temperature (*results not shown*). Hence, thermal-responsive self-healing for PVA/C16 blend hydrogels was evaluated in cut samples maintained in water at *T* = 60 °C for 24 and 72 h (Figure 5).

**Figure 5:**
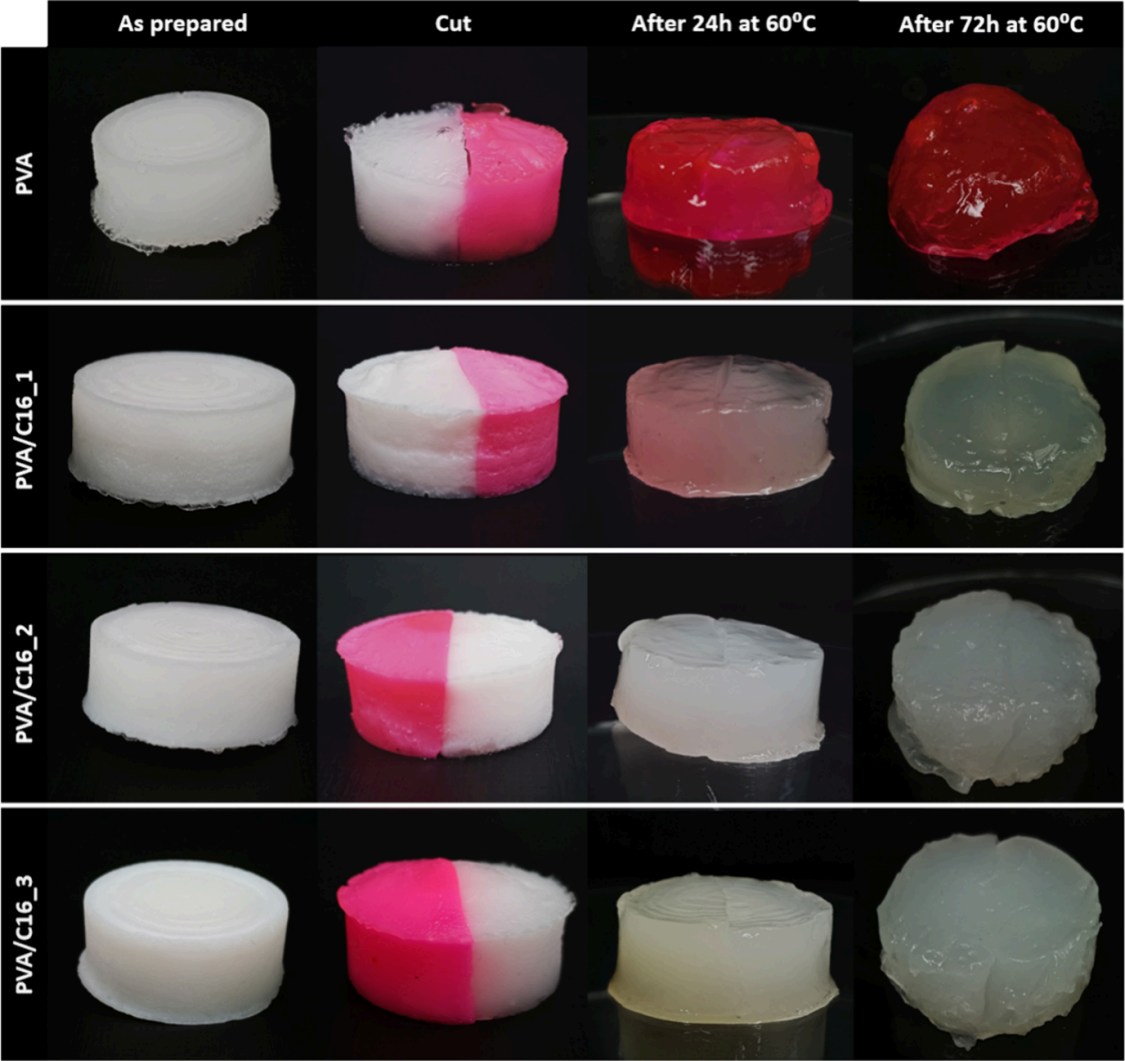
Self-healing tests carried out on “as prepared” PVA hydrogels and their blends with C16.

An annealing temperature of 60 °C was chosen for self-healing tests as this is the onset *T*_m_ of PVA crystals in the hydrogels, as determined by DSC experiments (Figure 3). Unlike PVA hydrogels, which turned pink after the self-healing test because of the retention of the hydrophilic pink dye within the hydrogel, PVA/C16 blend hydrogels retained their initial white opaque appearance after the self-healing test. This difference is attributed to the presence of hydrophobic C16 domains within the PVA/C16 blend hydrogels. In contrast to the neat PVA hydrogel, which exhibited self-healing capacity after the thermal treatment for 24 and 72 h, the self-healing capacity of blend hydrogels was only partial but improved with more extended thermal treatment time. Specifically, after 24 h, the gel could be easily separated into two halves with a slight pull. In contrast, after 72 h, it remained healed, in agreement with results in the literature^31^ that showed that chain diffusion and formation of new hydrogen bonds are highly dependent on time; nonetheless, the original cut was still visible. Semiquantitative self-healing tests (Figure S3 and Videos S1, S2, S3, and S4, SI) demonstrated that PVA/C16 blend hydrogels could lift and sustain 368 and 124 g, while the neat PVA hydrogel lifted only 124 g, highlighting the positive effect of hexadecylamine on self-healing performance.

Interestingly, PVA/C16 blend hydrogels maintained their dimensional stability after the thermal self-healing tests, highlighting the toughening effect of C16 incorporation, as it will be corroborated through oscillatory rheological experiments that will be shown below. Self-healing in PVA hydrogels obtained through F-T cycles has been explained based on polymer chain diffusion and the formation of new hydrogen bonds at the fracture surface.^9^ The lower self-healing capacity exhibited by PVA/C16 blend hydrogels can be explained by the higher crystallinity exhibited by these hydrogels compared to neat PVA hydrogels, as demonstrated by DSC and WAXS measurements. This not only reduces the availability of hydroxyl groups for hydrogen bonds formation but also hinders chain diffusion/reentanglement with respect to neat PVA hydrogels.

Oscillatory rheological experiments were carried out as a function of frequency and temperature to determine the rheological properties of annealed hydrogel samples. The results were compared to those exhibited by “as prepared” hydrogel samples (Figure 6). Oscillatory frequency tests revealed that both the neat PVA hydrogel and the PVA/C16_1 sample exhibit an elastic modulus (*G*′) of approximately 4000 Pa within experimental error. This value significantly increased with the addition of C16, reaching *G*′ values of 7379 ± 91 Pa for the PVA/C16_2 sample and 8803 ± 37 Pa for the PVA/C16_3 sample (Figure 6a). All samples exhibit the typical behavior of a gel, where *G*′ (elastic modulus) is consistently higher than *G*″ (viscous modulus) across the entire experimental frequency range, with both moduli remaining independent of frequency that has also been reported for F-T PVA hydrogels.^32^ Upon thermal annealing, a decrease in *G*′ is observed for all samples, particularly pronounced in the neat PVA hydrogel due to partial gel melting that also results in some dependence of *G*′ and *G*″ as a function of frequency, showing an increased elastic modulus at high frequencies (Figure 6b).

**Figure 6:**
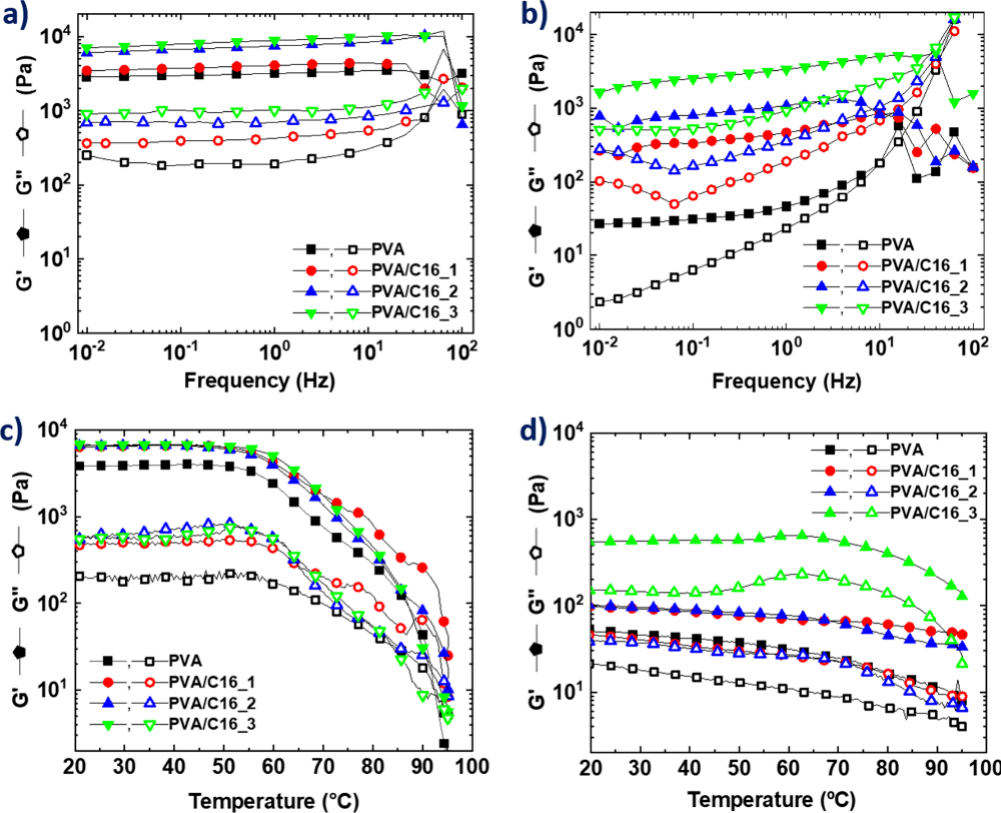
Oscillatory frequency test carried out for (a) “as prepared” hydrogels and (b) “annealed” hydrogels. Oscillatory temperature test carried out for (c) “as prepared” hydrogels and (d) “annealed” hydrogels.

Oscillatory temperature sweeps reveal a similar trend, confirming that adding C16 increases the elastic modulus at temperatures below *T*_m_ because crystals act as physical cross-linking points in the blend hydrogels. Despite the variations in composition, all samples preserved a gel structure up to 60 °C, as evidenced by the *G*′ value being greater than the *G”* and both moduli remaining constant over a broad temperature range. The transition from gel-to-sol occurred when both moduli decreased at temperatures above 60 °C, roughly coinciding with the endothermic peak observed in the DSC corresponding to the melting of PVA crystals. This overlap suggests that the crystallites are responsible for maintaining the three-dimensional gel network structure.^33^ (Figure 6c).

However, after thermal annealing, the elastic moduli of all samples decreased, and *T*_m_ shifted to higher temperatures. For example, in the case of the PVA/C16_3 sample, *T*_m_ shifts from 60 °C in the “as prepared” sample to *T*_m_ = 70 °C for the annealed sample (Figure 6d). This shift suggests that thermal annealing induces structural rearrangements within the hydrogel network, likely leading to increased crystallinity or enhanced interactions among polymer chains. These findings align with the DSC results presented in the Supporting Information (Figure S4, SI), where a similar increase in *T*_m_ was observed for PVA/C16_3 subjected to thermal annealing. This consistency between rheological and thermal analysis further supports that thermal annealing at temperatures close to *T*_m_ increases physical cross-linking and crystalline domains within the blend hydrogels. Such an increase enhances mechanical and thermal properties, thereby imparting self-healing properties, as will be demonstrated below.

This idea is consistent with the observations made by Song et al., who determined a critical annealing temperature of 74 °C. When annealing occurs above this temperature, the gels exhibit a double structure: a hard crystalline network and a soft amorphous network, both of which affect the mechanical response. On the other hand, if the temperature is below 74 °C, the mechanical response depends exclusively on the soft amorphous network.^4^

In addition, all the samples under study showed consistent adhesive behavior on various substrates with varying surface energies after being annealed at *T* = 60 °C for 72 h (Figure 7a). In contrast, the “as prepared” samples, without any annealing treatment, exhibited good adhesion only to glass (Figure S5, SI). In addition, unlike neat PVA hydrogels, blend hydrogels retained their mechanical stability upon being adhered to a glass surface (5 g) in a static shear test (Figure 7b).

**Figure 7:**
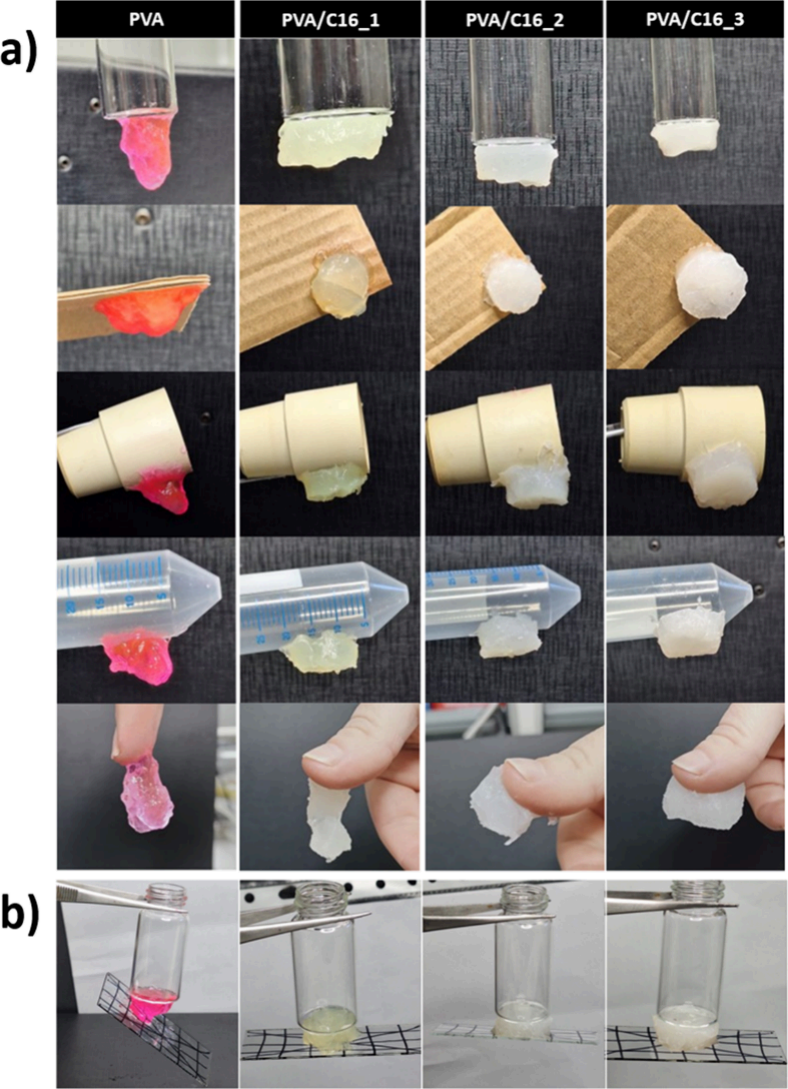
(a) PVA/C16 blend hydrogels are capable of adhering to various substrate surfaces, including glass, cardboard, rubber, and polypropylene, after undergoing thermal treatment at *T* = 60 °C for 72 h; (b) PVA/C16 blend hydrogels maintain their dimensional stability when adhered to a glass slide under a 5 g load.

Figure 8 shows probe-tack experiments carried out on neat PVA and PVA/C16_3 blend hydrogels. During the experiment, data on the normal stress σ and the distance between the two plates are collected. From these, a stress (σ)-strain (ε) curve is obtained. The strain values are calculated using eq 2, where *h*_0_ is the initial thickness of the material. The stress–strain curve (Figure 8) allows obtaining the maximum stress σ_max_ and the work of adhesion *W*_adhesion_, which is calculated using eq 3.^34^

$$\varepsilon =(h(t)-h_{0})/h_{0}$$ 2

$$W_{\mathrm{adhesion}}=h_{0}\int_{0}^{\varepsilon_{\max}}\sigma (\varepsilon )d\varepsilon$$ 3

**Figure 8:**
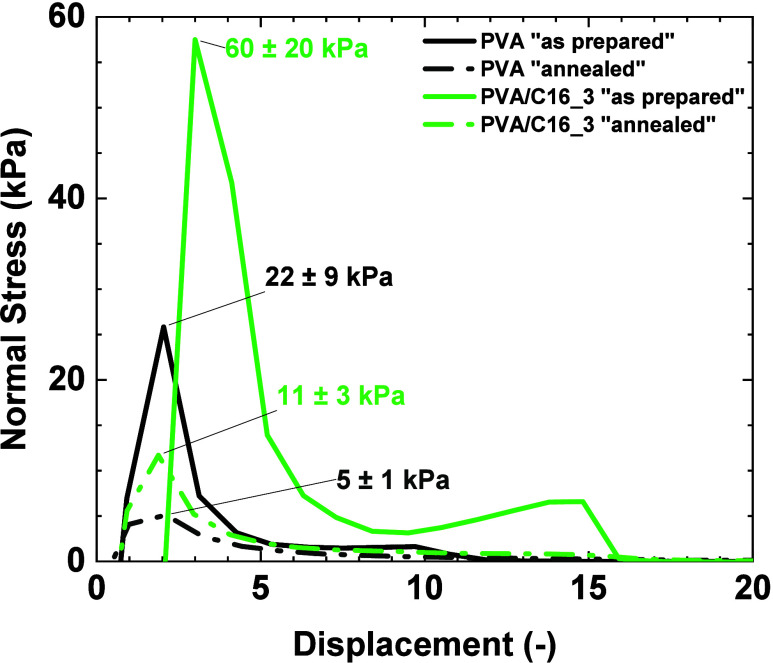
Stress–displacement curves of “as prepared” and “annealed” PVA and PVA/C16_3 hydrogels obtained from probe-tack experiments carried out at room temperature using glass as a substrate. The σmax value (kPa) for each of the samples is shown in the graph.

The behavior of the material after the σ_max_ value allows us to distinguish between a viscous liquid and a viscoelastic solid. If the normal stress decreases abruptly, it is associated with a viscous liquid. Hydrogels exhibit this behavior after heat treatment. On the other hand, if the normal stress decreases more slowly, it is a viscoelastic solid, a behavior exhibited by gels before heat treatment.^35^ In general, hydrogels adhere through surface intermolecular interactions, such as hydrophobic, electrostatic, or hydrogen bonds. It is well-known that higher cross-linking provides better mechanical properties in PVA gels, but it also affects hydrogel adhesion because the hydroxyl groups used in cross-linking are not available to adhere to a substrate surface.^36^

As shown in Figure 7, the “as prepared” PVA/C16_3 blend hydrogel exhibits a σ_m__a__x_ value of 60 ± 20 kPa compared to the σ_m__a__x_ value of neat PVA hydrogel (22 ± 9 kPa), with values falling within the range reported in the literature for hydrogel systems ≈20 kPa.^37^ Upon thermal annealing, the σ_m__a__x_ value decreases significantly for both hydrogels; however, the annealed PVA/C16_3 blend hydrogel still displays a higher σ_m__a__x_ (11 ± 3 kPa) than the annealed neat PVA hydrogel (5 ± 1 kPa). These results suggest that the incorporation of hexadecylamine has a dual effect: enhancing thermal resistance and improving adhesion to the glass substrate.

Liu et al. demonstrate that the enlargement and orientation of PVA nanocrystalline domains enhances the interaction between them, which prevents crack propagation in the hydrogel structure and improves its mechanical properties. This is because a strain energy of approximately 50,000 kJ/mol is required to pull out a PVA chain from a crystalline domain and 6000 kJ/mol from an amorphous domain. Therefore, the interaction between the nanocrystalline domain and the glass requires approximately 70,000 kJ/mol.^37^

This explains what is observed in (Figure 8) of our study, where the presence of hexadecylamine promotes the formation of additional PVA crystals within the hydrogel matrix, providing greater adhesion than pure PVA.

## 4 Conclusions

This study found that blending hexadecylamine with PVA in solution, followed by applying freezing-thaw (F-T) cycles, induces PVA blend hydrogel formation with improved mechanical properties and additional functionalities such as self-healing and adhesion properties. The phase separation induced by adding a nonmiscible compound to PVA in water significantly affected the degree of crystallinity of the PVA/C16 blend hydrogels, as confirmed by DSC and temperature-dependent WAXS experiments. The DSC analysis reveals distinct differences in thermal behavior between neat PVA hydrogels and PVA/C16 blend hydrogels. While neat PVA hydrogels exhibit negligible crystallinity, incorporating C16 significantly enhances PVA crystallization, leading to well-defined thermal transitions. Additionally, partially drying the gels further stabilizes the crystalline domains. These findings highlight the critical role of C16 in modifying the phase behavior of PVA-based hydrogels, confirmed by WAXS and DSC results that provide strong evidence that incorporating C16 promotes the formation of additional PVA crystals within the hydrogel matrix.

While water content influences the degree of crystallinity, the presence of C16 further stabilizes the PVA semicrystalline network, leading to an overall increase in structural order. Simultaneously, PVA/C16 blend hydrogels exhibit adhesive properties, particularly after thermal annealing at temperatures near the gel melting point. At room temperature, a proof-of-concept experiment was conducted to quantify the adhesive strength of both neat PVA and PVA/C16_3 hydrogels. Results showed that adhesion to polar surfaces, such as glass, doubled in the case of the PVA/C16_3 hydrogel compared to neat PVA. This enhancement in adhesive strength can be attributed to the incorporation of polar amine groups within the hydrogel network, which likely promotes stronger interactions with polar substrates through hydrogen bonding or electrostatic interactions.

The materials reported herein pave the way for developing functional hydrogels with adhesive and self-healing properties. This is achieved through a straightforward and efficient methodology based on crystallization-driven gelation by blending an immiscible component. Furthermore, the versatility of this strategy allows for fine-tuning of mechanical, adhesive, and self-healing properties by modifying the composition or processing conditions, making these hydrogels promising candidates for advanced applications in bioelectronics, soft sensors, and controlled drug delivery systems.

## Supplementary Material











## Data Availability

Raw data corresponding to wide-angle X-ray scattering (WAXS), Raman spectroscopy, differential scanning calorimetry (DSC), and rheological measurements can be found at: 10.20350/digitalCSIC/17401.

## Notes

Figure S1: macroscopic appearance of the 10% (w/w) PVA aqueous solutions and PVA/C16 blend solutions prior to gel formation; Figure S2: ATR- FTIR spectra of freeze-dried PVA/C16_3 blend hydrogel, neat PVA, and hexadecylamine (C16); Figure S3: (a) appearance of the hydrogels before and after the self-healing test and (b) weights used for the semiquantitative self-healing tests and Videos S1–S4; Figure S4: DSC analysis of PVA and PVA/C16_3 blend hydrogels, ‘as prepared’ and after annealing treatment (72 h at 60 °C); and Figure S5: images showing the adhesive character of “as prepared” hydrogels toward different surfaces (PDF)
Semiquantitative self-healing tests of PVA (MP4)
Semiquantitative self-healing tests of PVA/C16_1 (MP4)
Semiquantitative self-healing tests of PVA/C16_2 (MP4)
Semiquantitative self-healing tests of PVA/C16_3 (MP4)
