# Supplementary material for: Hexadecylamine Addition Promotes Crystallization-Driven Functionalities in Freezing–Thaw PVA Hydrogels
Source: Macromolecules. 2025 Nov 28;58(23):12564–73. doi: 10.1021/acs.macromol.5c01534 (PMC12874647; doi:10.1021/acs.macromol.5c01534)
Supplement: Supplementary file 1 [file ma5c01534_si_001.pdf]

SUPPLEMENTARY INFORMATION

Hexadecylamine addition promotes crystallization-driven functionalities in freezing-thawing PVA hydrogels

Alexis Alvear-Jiménez[a,b], Mercedes Fernández[b], Alejandro J. Müller[b,c*], Rebeca Hernández[a*]

[a] Institute of Polymer Science and Technology ICTP-CSIC, Juan de la Cierva 3, 28006 Madrid, Spain.

[b] POLYMAT and Department of Polymers and Advanced Materials: Physics, Chemistry and Technology, Faculty of Chemistry, University of the Basque Country UPV/EHU, Paseo Manuel de Lardizabal, 3, 20018 Donostia-San Sebastián, Spain

[c] IKERBASQUE, Basque Foundation for Science, Plaza Euskadi 5, Bilbao, 48009, Spain

Corresponding authors: alejandrojesus.muller@ehu.es; rhernandez@ictp.csic.es

List of figures

Figure S 1. Macroscopic appearance of the 10% (w/w) PVA aqueous solutions and PVA/C16 blend solutions prior to gel formation.

Figure S2. ATR- FTIR spectra of freeze-dried PVA/C16_3 blend hydrogel, neat PVA and hexadecylamine (C16)

Figure S3. a) Appearance of the hydrogels before and after the self-healing test. b) Weights used for the semi-quantitative self-healing tests.

Figure S4. DSC analysis of PVA and PVA/C16_3 blend hydrogels, `as prepared´ and after annealing treatment (72h at 60 ^{0}C).

Figure S5. Images showing the adhesive character of "as prepared" hydrogels towards different surfaces.

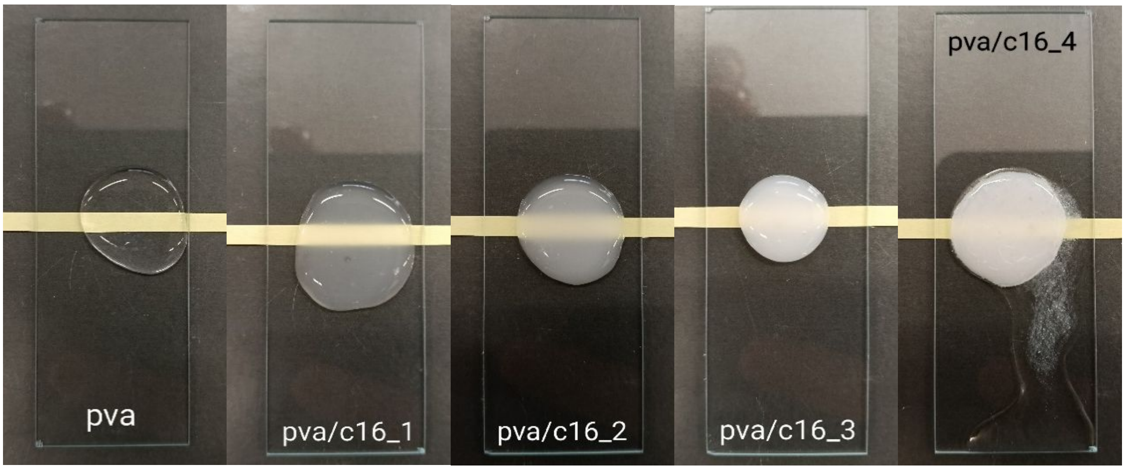

Figure S 1. Macroscopic appearance of the 10% (w/w) PVA aqueous solutions and PVA/C16 blend solutions prior to gel formation.

Attenuated Total Reflectance - Fourier Transform Infrared Spectroscopy (ATR-FTIR)

ATR-FTIR spectra were acquired using a PerkinElmer Spectrum One spectrometer equipped with an attenuated total reflectance (ATR) accessory. Measurements were carried out on freeze-dried samples over the spectral range of 4000–400 cm^{-1}, with a spectral resolution of 4 cm^{-1} and averaging 30 scans per sample to improve the signal-to-noise ratio. All spectra were subjected to ATR correction and baseline normalization prior to analysis to ensure consistency and comparability among samples.

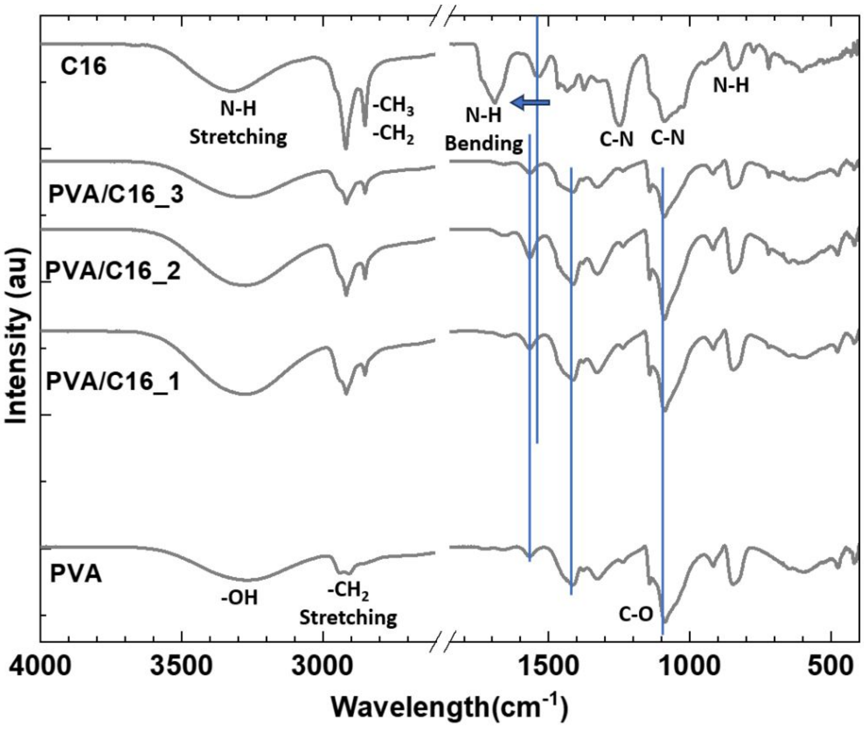

Figure S2. ATR- FTIR spectra of freeze-dried PVA/C16_3 blend hydrogel, neat PVA and hexadecylamine (C16)

Self-healing Test

The gels were cut horizontally and then immersed in a thermostat to control the temperature and prevent water loss. After 72 hours, the gels were cooled to room temperature, and a metal hook was inserted into each half to allow the gels to be hung with the weights and to verify whether they were able to lift and sustain the selected loads.

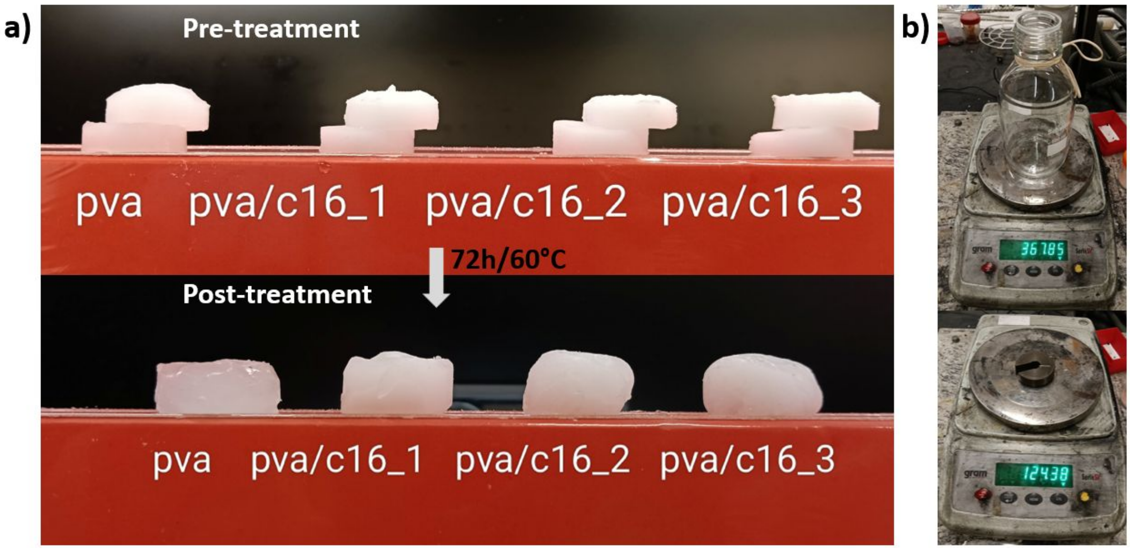

Figure S3. a) Appearance of the hydrogels before and after the self-healing test. b) Weights used for the semi-quantitative self-healing tests.

Semi-quantitative self-healing tests (Videos S1–S4) demonstrated that PVA/C16 blend hydrogels could lift and sustain 368 g and 124 g, while the neat PVA hydrogel lifted only 124 g, highlighting the positive effect of hexadecylamine on self-healing performance.

VIDEO S1: PVA
VIDEO S2: PVA/C16_1
VIDEO S3: PVA/C16_2
VIDEO S4: PVA/C16_3

Differential Scanning Calorimetry (DSC)

Differential Scanning Calorimetry (DSC) analyses were performed using a PerkinElmer Pyris DSC 8000 system equipped with an Intracooler 2P, operating under a continuous flow of ultrahigh-purity nitrogen. Approximately 15 mg of each sample were accurately weighed and hermetically sealed in standard PerkinElmer aluminum pans. An empty hermetic pan was used as a reference. Thermal scans were conducted over a temperature range of 20 to 90 °C at a constant heating rate of 5 °C/min. Analyses were performed on both “as-prepared” hydrogels and hydrogels subjected to an annealing treatment (72 hours at 60 °C).

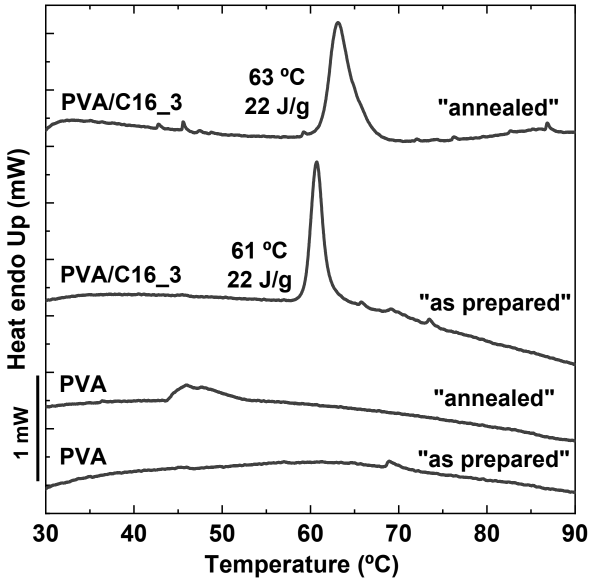

Figure S4. DSC analysis of PVA and PVA/C16_3 blend hydrogels, `as prepared´ and after annealing treatment (72h at 60 ^{0}C).

Determination of adhesive properties

As-prepared" hydrogels, including neat PVA hydrogels and PVA/C16_X blend hydrogels that had not undergone annealing treatment (i.e., 72 hours at 60 °C), were used in qualitative adhesion tests to evaluate their adhesive behavior on various substrates. The tests were conducted under ambient conditions, and the performance was qualitatively evaluated based on visual inspection and manual detachment resistance.

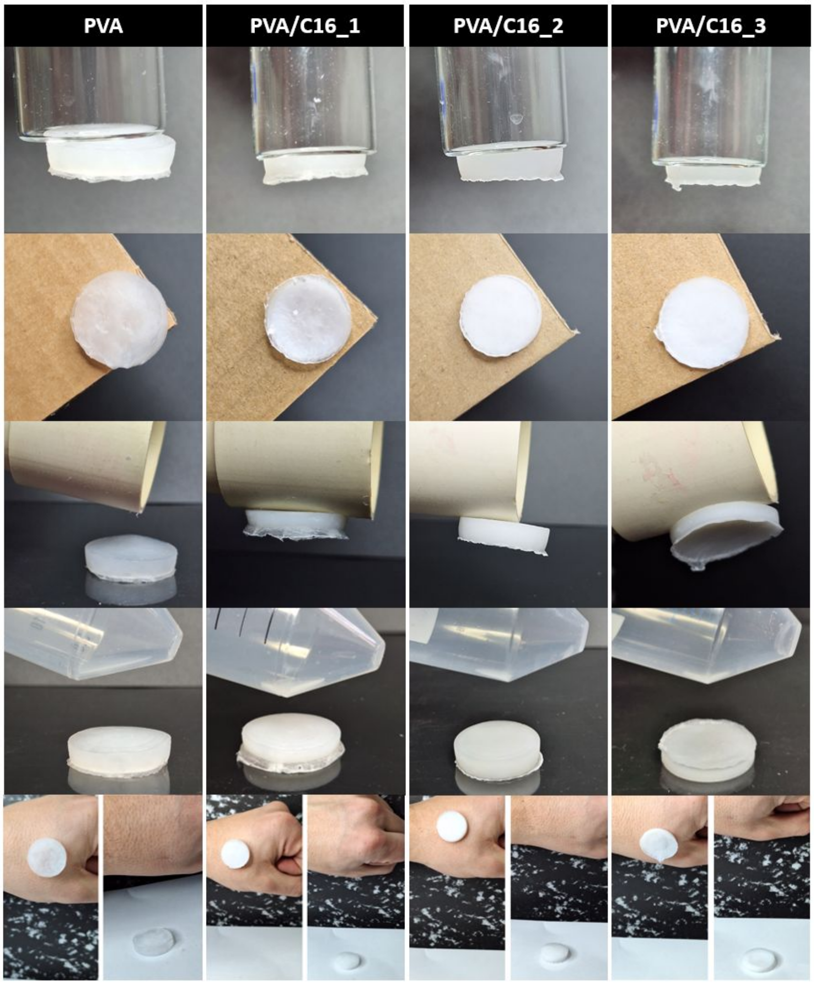

Figure S5. Images showing the adhesive character of "as prepared" hydrogels towards different surfaces.
